# A Consistent PCR-RFLP Assay Based on ITS-2 Ribosomal DNA for Differentiation of *Fasciola* Species

**Published:** 2013-12

**Authors:** Reza Shafiei, Bahador Sarkari, Abdolali Moshfe

**Affiliations:** 1 Department of Parasitology and Mycology, School of Medicine, Shiraz University of Medical Sciences, Shiraz, Iran; 2 Basic Sciences in Infectious Diseases Research Center, Shiraz University of Medical Sciences, Shiraz, Iran; 3 Cellular and Molecular Research Center, Yasuj University of Medical Sciences, Yasuj, Iran

**Keywords:** Differentiation, *Fasciola*, Restriction enzymes, RFLP

## Abstract

***Objective(s):*** Fascioliasis is a zoonotic parasitic disease caused by liver fluke species of *Fasciola hepatica *and* Fasciola gigantica*. Differentiation of these two species, based on their morphological characteristics, is difficult. The current study aimed to use PCR-RFLP assay to distinguish between *F. hepatica* and *F. gigantica*, based on profiles of RFLP, produced by effect of endonucleases on ITS2 of the ribosomal DNA genes from these two species.

***Materials and Methods:*** Adult *Fasciola *spp. were isolated from bile duct of naturally infected animals. The species of *Fasciola* were confirmed by sequencing the 505 bp region of the ITS2 gene in the isolates. By running the sequences of the samples in NEBcutter, suitable restriction enzymes (*Msp*I and *Kpn*I) were selected. Eight *F. gigantica* and eighteen *F. hepatica* samples were evaluated.

***Results:*** While RFLP pattern with *Msp*I produced a profile by which it was difficult to differentiate these two species, *Kpn*I along with *Msp*I, produced a consistent pattern of a 231, 212 and 93 bp fragments in *F. hepatica*. This pattern was not seen in *F. gigantica*.

***Conclusion:*** Findings of this study demonstrated that RFLP with *Kpn*I and *Msp*I produce a suitable pattern which simply differentiates *F. hepatica *from* F. gigantica*.

## Introduction

Fascioliasis is a zoonotic parasitic disease caused by the liver fluke species of the genus *Fasciola*. *F. hepatica *and *F. gigantica *are two species which infect human and animals. *F. hepatica* has a worldwide distribution and both species are present in the tropical and subtropical regions of Africa and Asia (1).

Differentiation of these two species, based on their morphological characteristics, is difficult. The differences in the intermediate hosts, control strategies, transmission patterns and also epidemiological characteristics which overlap in some area indicate that the proper differentiation of *F. hepatica* and *F. gigantica* infections in human or animals is crucial (2).

Due to the limitations of morphological methods, several molecular approaches, using different molecular targets, have been developed for the differentiation of *F. hepatica* and *F. gigantica* (3). 

Several DNA-based approaches have been used for differentiation of *Fasciola* species (4-7). Among them, sequencing of the first and the second internal transcribed spacers (ITS-1 and ITS-2) of ribosomal DNA and mitochondrial DNA (mtDNA) provided reliable genetic markers for species-level identification of *Fasciola* species (3). ITS-2 sequence is located between the 5.8S and 28S coding regions of rDNA that are highly conserved and have few inter-specific nucleotides useful for genetic characterization and identification in both *F. hepatica* and *F. gigantica* (8-9).

Although sequencing of ribosomal or mitochondrial DNA which shows the differences between the nucleotides of the desired gene, offers reliable methods for differentiation of *Fasciola* species, RFLP-based approaches provide a relatively simple, cost-effective and appropriate method for differential diagnosis of *F. hepatica* and *F. gigantica*. In line with this, different restriction enzymes and target genes have been used (2). The current study was performed to examine the utility of a PCR-RFLP assay for differentiation of *F. hepatica* and *F. gigantica*, based on RFLP profiles, produced by the effect of endonucleases on ITS2 of the ribosomal DNA genes from these two species.

## Materials and Methods

***Parasite***

Adult *Fasciola *spp. were isolated from bile duct of naturally infected sheep, goat and cattle at the slaughterhouses from various regions of Kohgiluyeh and Boyer-Ahmad province in Iran, where human cases of fasciolosis has been recently reported (10). Flukes were washed extensively in PBS (37°C) and subsequently fixed in 70% ethanol and maintained at 4°C for several weeks until used. 

***DNA extraction and polymerase chain reaction (PCR)***

For genomic DNA extraction, a portion of the apical and lateral zone of adult flukes was removed and crushed. DNA from the crushed materials was extracted with phenol–chloroform method. Briefly, 500 μl of lysis buffer and 8 μl of proteinase K was added to the sample and incubated overnight at 37°C. Afterward, 100 μl of phenol–chloroform was added and centrifuged at 1000 g for 10 min at 25°C. Top aqueous phase was removed and absolute alcohol was used to precipitate the DNA. Extracted DNA was diluted in double distilled water and preserved at 4°C until used.

Amplification of the DNA was performed as described by Itagaki *et al* (2005) using a pair primer to amplify a 505 bp region of the ITS2 sequence (11). PCR reaction contained 3 μl of DNA solution, 0.25 μl of Taq DNA polymerase (Cinnagen, Iran), 2.5 μl of 10x PCR buffer, 1 μl of MgCl_2_, 1 μl of each primers (Forward: 5′-TGTGTCGATGAAGAGCGCAG-3′ and Reverse: 5′-TGGTTAGTTTCTTTTCCTCCGC-3′), 1 μl of dNTPs and 15.5 μl of DDW. The DNA product was sequenced and *Fasciola* species were identified.

***Restriction fragment length polymorphism (RFLP)***

**Figure 1:**
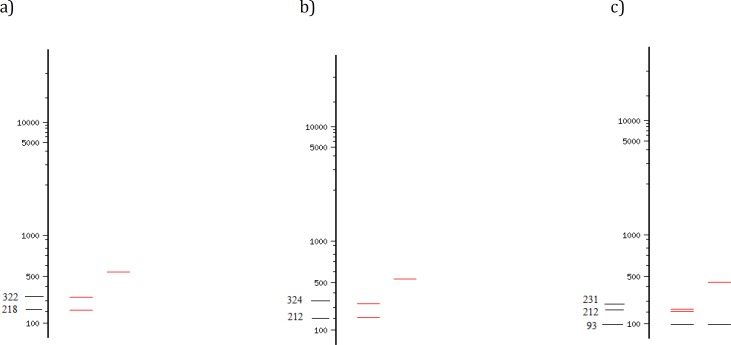
Cutting sites of *Msp*I restriction enzyme on a) *Fasciola gigantica, * b) *F. hepatica,* C) cutting sites of *Kpn*I on *F. hepatica.* *F. gigantica *has no cutting sites for* Kpn*I

After sequencing, using NEBcutter V2.0 software (12), the cutting sites of commercially available restriction enzymes on ITS2 sequences of *F. hepatica* and *F. gigantica* were assessed (Figure 1). *Msp*I and *Kpn*I were selected as the enzymes which might produce the most informative profile. For RFLP, a total volume of 20 μl, including 10 μl of ITS2 PCR product was added with 1 μl of either *Msp*I *or Kpn*I, 4 μl of 10x Tango buffer (Fermentas, Lithuania) and 4 μl of DD-H_2_O. The tubes were incubated at 37°C for 12 hr, according to the manufacturer instruction to ensure full cutting of fragments. For analyzing the digestion products, 15 μl of each product in addition to 2 μl of loading buffer were run in 2% gel electrophoresis. 

## Results

**Figure 2:**
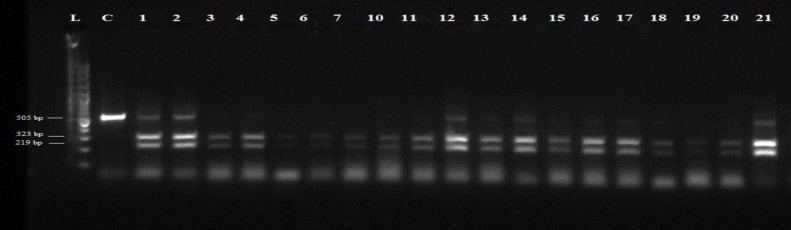
RFLP pattern of PCR products of *F. hepatica* and *F. gigantica* digested by *Msp*I. L) 100-bp DNA ladder, C) undigested PCR product for control, lane 1, 2, 4, 10, 12, 14, 15 and 19 are *F. gigantica*; lane 3, 5-7, 11, 13, 16-18, 20 and 21 are *F. hepatica*

**Figure 3:**
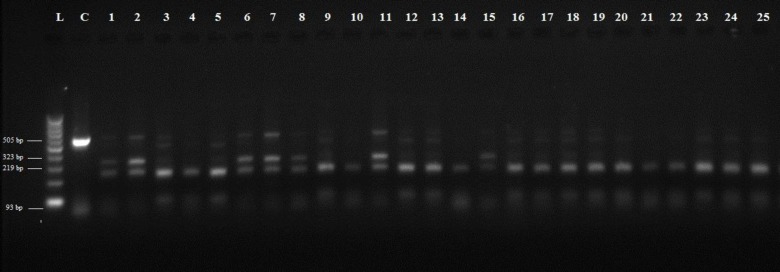
RFLP pattern of PCR products of *F. hepatica* and *F. gigantica* digested by *Kpn*I and *Msp*I. L) 100-bp DNA ladder, C) undigested PCR product for control; lane 1, 2, 6, 7, 8, 11, 15 are *F. gigantica* and the rest are *F. hepatica*

The species of *Fasciola* were confirmed by sequencing the 505 bp region of the ITS2 gene in the isolates. All amplified products of both *F. hepatica and F. gigantica *were digested with the *Msp*I restriction endonuclease. Eight *F. gigantica* and eighteen *F. hepatica* samples were evaluated. RFLP pattern with *Msp*I produced a 212 and 324 bp fragments for *F. hepatica* and 218 and 322 bp for *F. gigantica *(Figure 2)*. *Based on these profiles, it was quite difficult to differentiate these two species. Using *Kpn*I along with* Msp*I (double digestion), a consistent pattern was found where *Kpn*I cut ITS2 fragment of *F. hepatica *and produced a 231, 212 and 93 bp fragments (Figure 3). This pattern was not seen in *F. gigantica* (Figure 3).

## Discussion

Differentiation of *Fasciola* species is crucial for proper implementation of any control measurement about both human and animal fascioliasis (1). Molecular markers based on DNA analysis have been employed for genetic characterization and identification of parasites especially helminthes (13, 14). In the present study, a rapid and simple method of RFLP assay, based on the partial rDNA of ITS2 gene, was utilized for the accurate differentiation and identification of *Fasciola *species.

Restriction enzymes are powerful and simple approaches toward the characterization of parasite species based on differences in their genomes. These methods have been used for differentiation among *Fasciola *species based on the profiles generated by the effects of endonucleases on ITS genes of these parasites (4, 5). In a study in China, effect of *Hsp92*II or *Rca*I on ITS2 region, *Fasciola *spp. were differentiated from one another by their unique restriction patterns (8). Isolates of Chinese *Fasciola* produced a mixture of patterns in two *Fasciola* species. Huang *et al* (2004) used the restriction endonucleases, *Hsp92*II and *Rca*I, on ITS2 region for molecular differentiation of *Fasciola *spp. and showed that *Hsp92*II produces different profiles in different isolates from different hosts. They reported that *Hsp92*II is better than *Rca*I for differentiating between these two species (8). 

*Fasciola* species have been traditionally classified based on their morphological features, such as width and body length. Because of the size variations of these two species, the discrepancy of morphological features, and the presence of intermediate forms, it is difficult to distinguish the two species, solely based on these characteristics. RFLP is a powerful approach for discriminating these two species. RFLP pattern have been used for characterization of *Fasciola* species and also other organisms in Iran (15-18). Karimi (2008) showed that in 18S DNA region, *Bfr*I restriction enzyme produce similar profile for both *F. hepatica* and *F. gigantica* whereas *Dra*I generates different patterns for two species of *Fasciola* (15). Rokni *et al* (2010) used *Tas*I restriction enzyme for ITS1 region which properly differentiated *F. hepatica* from *F. gigantica* (2). In another study, Saki *et al* (2011) showed that *Ava*II and *Dra*II restriction enzymes in 28S DNA appropriately differentiate these two species (16). However, Ghavami *et al* (2009) showed that the pattern of restriction digestion in ITS2 sequence of *Fasciola* samples which was seen with *BamH*I and *Pag*I restriction enzymes at the nucleotide positions of 230, 340 and 341 bp are specific to *F. hepatica* species and has no effect on *F. gigantica* (17). 

## Conclusion

In the current study, considering the sequences of ITS2 of *F. hepatica* and *F. gigantica*, two endonucleases, *Msp*I and *Kpn*I, were used to differentiate *Fasciola *spp. For *Msp*I*, *the RFLP profiles of two species were very similar while for *Kpn*I the profile was quite different and this enzyme was able to cut the ITS2 of two species of *Fasciola* at different sites which may be utilized for the differentiation of two species. The evaluation of the *Fasciola *spp. in the areas where two species of the fluke coexist is important and this simple RFLP can be used for discerning these two species.
